# Effects of *Clostridium butyricum* on Production Performance and Bone Development of Laying Hens

**DOI:** 10.3390/vetsci11040160

**Published:** 2024-04-01

**Authors:** Jiaqi Huang, Lulu Cui, Hai Lin, Mengze Song, Shuhong Sun

**Affiliations:** 1 Department of Preventive Veterinary Medicine, College of Animal Science and Technology, Shandong Agricultural University, Tai’an 271018, China; 2020110516@sdau.edu.cn (J.H.); 2021010090@sdau.edu.cn (L.C.); 2 Shandong Provincial Key Laboratory of Animal Biotechnology and Disease Control and Prevention, Shandong Agricultural University, Tai’an 271018, China; hailin@sdau.edu.cn

**Keywords:** *Clostridium butyricum*, laying hens, bone, average egg weight, production performance, egg quality

## Abstract

**Simple Summary:**

Probiotics are safe, inexpensive, and effective feed additives, and *Clostridium butyricum* (CB) has been reported to regulate bone health in addition to having conventional probiotic effects. We found that CB had little effect on the body weight and feed intake of laying hens. Feed additions of 10^8^ and 10^9^ CFU/kg CB can significantly increase the tibia index and bone mineral density of four-week-old green-shell laying hens. Feed additions of 10^7^ and 10^8^ CFU/kg CB can significantly increase the average egg weight, eggshell weight, and tibia index of 26-week-old Luhua laying hens, but 10^7^ CFU/kg CB will reduce the egg production rate. Adding 10^8^ CFU/kg CB to feed can significantly increase the average egg weight, eggshell weight, and tibia bending strength of 40-week-old Hy-line Brown laying hens. In summary, adding 10^8^ CFU/kg CB is beneficial to the bone and production health of laying hens.

**Abstract:**

Probiotics are safe, inexpensive, and effective feed additives, and *Clostridium butyricum* (CB) has been reported to regulate bone health in addition to having conventional probiotic effects. The bone health of laying hens is closely related to their production performance. Here, we investigated the effects of CB supplementation on the bone health and performance of laying hens. We added CB to the feed of green-shell laying hens, Luhua laying hens, and Hy-line Brown laying hens and examined changes in body weight, feed intake, egg production performance, and egg quality to determine the impact of CB on production performance. The impact of CB on the bones of laying hens was determined by analyzing the bone index, bone bending strength, bone calcium and phosphorus content, and bone mineral density. The study found that CB had little effect on the body weight and feed intake of laying hens. Feed additions of 10^8^ and 10^9^ CFU/kg CB can significantly increase the tibia index and bone mineral density of four-week-old green-shell laying hens. Feed additions of 10^7^ and 10^8^ CFU/kg CB can significantly increase the average egg weight, eggshell weight, and tibia index of 26-week-old Luhua laying hens, but 10^7^ CFU/kg CB will reduce the egg production rate. Adding 10^8^ CFU/kg CB to feed can significantly increase the average egg weight, eggshell weight, and tibia bending strength of 40-week-old Hy-line Brown laying hens. In summary, adding 10^8^ CFU/kg CB is beneficial to the bone and production health of laying hens.

## 1. Introduction

Poultry provides energy, protein, and essential micronutrients to humans [1]. To meet the growing market demand for meat and eggs, feed additives are gaining im-portance in the poultry industry due to their wide-ranging beneficial effects, such as promoting growth and production, enhancing immunity, and protecting health [2,3,4]. Probiotics, including *Bacillus*, *Lactobacillus*, *Lactococcus*, *Streptococcus*, *Bifidobacterium*, and yeast, are safe, inexpensive, and effective feed additives that have been widely used in poultry farming [5,6].

*Clostridium butyricum* (CB), a butyrate-producing, spore-forming anaerobic bacterium, is found in a wide variety of environments, including soil, cultured milk products, and vegetables [7]. CB consumes undigested dietary fiber and produces short-chain fatty acids (SCFAs), particularly butyrate and acetate [8]. SCFAs have numerous important effects on host health, including the regulation of intestinal immune homeostasis, improvement of gastrointestinal barrier function, and reduction of inflammation [9], making CB a promising probiotic. In addition, CB also has biological characteristics such as high temperature resistance, acid resistance, and antibiotic sensitivity, which are suitable for feed additives [10]. Xiang’s research found that CB reduced the feed intake of Lohmann pink laying hens (180 days), improved the feed conversion ratio, eggshell strength, and albumen index, and benefitted gut health [11]. Zhan’s research found that CB improved egg production and eggshell strength and benefitted the immune function and antioxidant capacity of Jinghong-1 strain laying hens (336 days) [12]. There are also studies showing that CB can reduce fat deposition in the livers of Hy-line Brown laying hens (420 days) [13] and lower the production of sandpaper-shelled eggs in Hy-line Brown laying hens (450 days) [14].

The egg production process of laying hens is inseparable from bone health. Several studies have confirmed the positive effects of CB [15,16] and butyrate [17] on bone health, but there are only limited studies that have been conducted on poultry. This study aims to explore the effects of CB on the production performance and bone development of laying hens, which will lay the foundation for better application of CB in the poultry egg industry.

## 2. Materials and Methods

All procedures used in this study were approved by the Animal Care Committee of Shandong Agricultural University (Shandong, China) and carried out in accordance with the guidelines for experimental animals published by the Ministry of Science and Technology (Beijing, China).

### 2.1. Bacterial Strains

CB was obtained from the Dalian Sanyi Animal Medicine Company (Dalian, China).

### 2.2. Experimental Design

Green-shell laying hens: This experiment adopted a single-factor, completely random design. A total of 240 one-day-old healthy green-shell laying hens with similar body weights were randomly divided into three treatment groups; each treatment group had eight replicates, and each replicate had 10 chickens. The experiment started when the chickens were one day old. After the experiment started, the control group was fed the basal diet; the 10^8^ CB group was fed the basal diet supplemented with 1 × 10^8^ CFU/kg CB; and the 10^9^ CB group was fed the basal diet supplemented with 1 × 10^9^ CFU/kg CB (Table 1). Chicken feed was prepared every two days, and the chickens ate and drank freely. The experiment period lasted four weeks. Body weight and feed intake were measured regularly every two days. During the fourth week, one chicken was selected from each replicate, and the liver, spleen, thymus, and bursa of Fabricius were collected and weighed to determine the organ index. The length of the duodenum, jejunum, ileum, and cecum were measured to assess intestinal development. Both tibia and femur were collected to detect bone index, bone bending strength, bone calcium and phosphorus content, and bone metabolism-related cytokines to evaluate bone development.

Luhua laying hens: This experiment adopted a single-factor, completely random design [2]. A total of 180 126-day-old healthy Luhua chickens with similar body weights were randomly divided into three treatment groups; each treatment group had six replicates, and each replicate had 10 chickens. After the experiment started, the control group was fed the basal diet; the 10^7^ CB group was fed the basal diet supplemented with 1 × 10^7^ CFU/kg CB; and the 10^8^ CB group was fed the basal diet supplemented with 1 × 10^8^ CFU/kg CB (Table 1). The experiment diet was configured once every two weeks, and the chickens had free access to food and water. The experiment lasted ten weeks, including a two-week pre-feeding period. Chickens were 140 days old when treated with CB. The number of eggs and egg weight of each replicate were recorded regularly every day. Eggs were collected in the second, fourth, sixth, and eighth weeks of the experiment to determine egg quality. At the end of the eighth week of the experiment, one chicken was selected from each replicate, and the tibia and femur were collected after euthanasia to detect bone development.

Hy-line Brown laying hens: This experiment adopted a single-factor, completely random design. A total of 48 224-day-old healthy Hy-line Brown laying hens with similar body weights were randomly divided into two treatment groups; each treatment group had six replicates, and each replicate had four chickens. After the start of the experiment, the control group was fed the basal diet, and the CB group was fed the basal diet supplemented with 1 × 10^8^ CFU/kg CB (Table 1). The experiment diet was configured once every two weeks, and the chickens had free access to food and water. The experiment lasted ten weeks, including a two-week pre-feeding period. Chickens were 238 days old when treated with CB. The number of eggs and egg weight of each replicate were recorded regularly every day, and the egg production rate and average egg weight were calculated. Eggs were collected in the second, fourth, sixth, and eighth weeks of the experiment to determine egg quality. At the end of the eighth week of the experiment, one chicken was selected from each replicate, and the tibia and femur were collected after euthanasia for detection of bone development.

### 2.3. Laying Performance

Egg production and egg weight were recorded daily, and feed consumption was recorded every two weeks. Average egg weight = egg weight/number of eggs laid. Average feed intake = feed consumption/days/number of chickens. Feed conversion rate = feed consumption/days/egg weight.

### 2.4. Egg Quality

Egg shape index: A vernier caliper was used to measure the value of the longitudinal and transverse diameters of the egg; egg shape index = transverse diameter/longitudinal diameter. Eggshell thickness: An ultrasonic thickness meter was used (ETG-1061, Robotmation, Japan) to measure the tip, blunt end, and middle points and take the average of the three values. Eggshell hardness: This was measured with an eggshell strength meter (EFG-0503, Robotmation, Japan). Albumen height, Haugh unit, egg yolk color: A multi-functional egg quality detector (EMT-5200, Robotmation, Tokyo, Japan) was used to detect the intact egg after removing the eggshell. The system detected, calculated, and reported values for yolk color (1 to 15 colors scale based on yolk color fan) and Haugh unit (Haugh unit = 100 × Log (H-1.7W^0.37^ + 7.57)), H: Albumen height, W: Egg weight. Yolk index: After measuring the Haugh unit of the egg, the egg yolk was removed to weigh and calculate the specific gravity of the egg yolk as follows: yolk index = weight of egg yolk/weight of egg × 100. Eggshell index: The eggshell was rinsed with clean water and dried. The eggshell weight was measured, and the eggshell gravity was calculated as follows: eggshell index = eggshell weight/egg weight × 100.

### 2.5. Bone

Bone bending strength: Bone bending strength was determined using three-point bending test to detect the maximum stress a bone could withstand before it broke [3]. Bone mineral density (BMD): Bone mineral density was measured at different spots in the tibia and femur using bone densitometer (InAlyzer, Baitai Technology Co., Ltd., Guangzhou, China). High-energy and low-energy two-layer X-rays were emitted through the X-ray tube; according to the different attenuation of the two layers of rays in different tissues, the corresponding BMD could be calculated by software. Calcium and phosphorus content: The tibia samples were treated with a mixture of alcohol and benzene at a ratio of 2:1 for 96 h for degreasing and then dried at 105 °C to maintain weight. The degreased bone samples were used to determine calcium and phosphorus content. Briefly, the degreased bones were first burned in a crucible heated by an electric ceramic furnace until they were carbonized, and then they were burned in a muffle furnace at 550 °C for 6 h. The calcium content was determined by potassium permanganate, and the phosphorus content was determined by the spectrophotometric [4].

### 2.6. Organ Index

After the dissection, the liver, spleen, bursa of Fabricius, thymus and bones were completely removed. Blood was blotted with filter paper, and the organ index was calculated as follows: organ index = organ weight/chicken weight ×100%

### 2.7. Bone RNA Extraction

The bone tissue sample (upper quarter of the tibia) was cut, ground in liquid nitrogen, placed in a 1.5 mL centrifuge tube with 1 mL TransZol, and homogenized in an ice bath with a tissue homogenizer. Then, it was centrifuged at 12,000 rpm for 15 min at 4 °C, and the supernatant was transferred to a new 1.5 mL centrifuge tube. Next, 200 μL of ice chloroform was added, and the mixture was vigorously shaken for 15 s, placed on ice for 20 min, and then centrifuged at 12,000 rpm and 4 °C for 20 min. The supernatant was transferred to a new 1.5 mL centrifuge tube, 250 μL of pre-cooled isopropanol and 250 μL of high salt solution (0.8 mol/L sodium citrate and 1.2 mol/L sodium chloride) were added, and the supernatant was iced for 20 min. Then, the supernatant was centrifuged at 4 °C and 12,000 rpm for 15 min and discarded. The RNA was washed with 1 mL of pre-cooled 75% ethanol and then centrifuged at 4 °C at 7500 rpm for 2 min. This was repeated three times; then, the centrifuge was left to stand until the ethanol evaporated. The RNA was then dissolved in DEPC water, and the sample was stored at −80 °C for later use [4].

### 2.8. Real-Time PCR Analyses

Total RNA was reverse-transcribed into complementary DNA (cDNA) using the RT First Strand cDNA Synthesis Kit (Roche, Basel, Switzerland). Quantitative real-time PCR (qRT-PCR) was performed using 2 × SYBR Green qPCR Master Mix (Servicebio, Wuhan, China) by an ABI QuantStudio 5 PCR machine (Applied Biosystems; Thermo, Waltham, MA, USA) with the following programs: 1 cycle at 95 °C for 10 min, 40 cycles at 95 °C for 15 s and 60 °C for 30 s. The primer sequences are provided in Table 2. Relative mRNA levels of specific genes were quantified using the 2^−ΔΔCt^ method values with *β-actin* as the reference gene.

### 2.9. Statistical Analysis

The data are expressed as means ± SD. The results were analyzed using one-way ANOVA in the Statistical Analysis Systems statistical software package (Version 8e; SAS Institute Inc., Cary, NC, USA). Differences between means were evaluated using Duncan’s significant difference tests. Means were considered significant at *p* < 0.05.

## 3. Results

### 3.1. Effect of CB on Green-Shell Laying Hens

**Figure 1:**
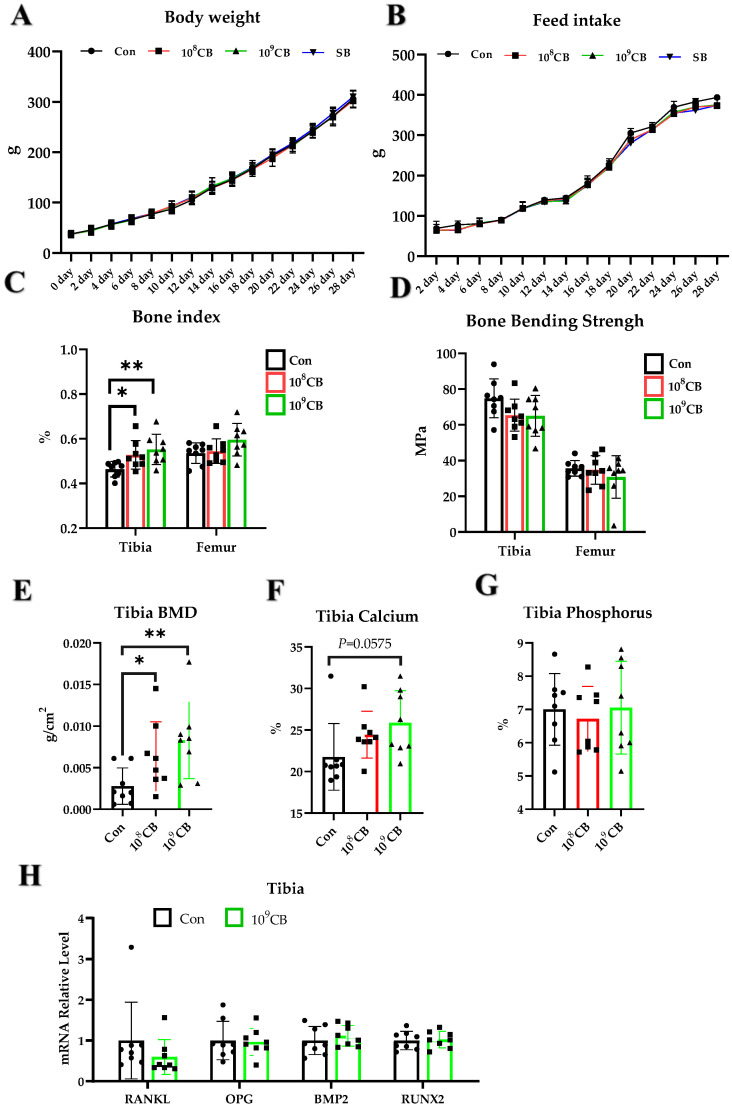
Effect of *Clostridium butyricum* (CB) on green-shell laying hens. (**A**,**B**) Changes in body weight and feed intake of laying hens over time. (**C**) Tibia and femur indexes. (**D**) Bending strength of tibia and femur. (**E**) Bone mineral density (BMD) of the tibia. (**F**) Tibial calcium content. (**G**) Tibia phosphorus content. (**H**) Tibial mRNA expression of *receptor activator of nuclear factor kappa-Β ligand* (*RANKL*), *osteoprotegerin* (*OPG*), *bone morphogenetic protein 2* (*BMP2*) and *runt-related transcription factor 2* (*RUNX2*). Con: Control group, fed basal diet; 10^8^ CB: basal diet supplemented with 1 × 10^8^ CFU/kg CB; 10^9^ CB: basal diet supplemented with 1 × 10^9^ CFU/kg CB. The data are presented as the mean ± SD. *, *p* < 0.05, **, *p* < 0.01.

The dietary supplementation of 10^8^ and 10^9^ CB had no significant effect (*p* > 0.05) on body weight (Figure 1A) and feed intake (Figure 1B). The overall period average feed intake of the 10^8^ CB and 10^9^ CB groups was significantly lower than that of the Con group (*p* < 0.05) (Table S1). The feed supplementation of 1 × 10^8^ and 1 × 10^9^ CB had no significant effect on the liver, spleen, thymus, bursa index, and duodenum, jejunum, ileum, cecum length (*p* > 0.05) (Table S2). Adding 1 × 10^8^ CFU/kg (*p* < 0.05) and 1 × 10^9^ CFU/kg (*p* < 0.01) CB in the feed can significantly increase the tibial index (Figure 1C). Adding 1 × 10^8^ and 1 × 10^9^ CB did not have a significant effect on the femur index (*p* > 0.05) (Figure 1C). Adding 1 × 10^8^ and 1 × 10^9^ CB did not have a significant effect on the bending strength of the tibia and femur (Figure 1D). Adding 1 × 10^8^ CFU/kg (*p* < 0.05) and 1 × 10^9^ CFU/kg (Figure 1C) CB in the feed can significantly increase the tibial BMD (Figure 1E). Adding 1 × 10^8^ CFU/kg (*p* > 0.05) and 1 × 10^9^ CFU/kg (*p* = 0.0575) CB had no significant effect on tibia calcium and phosphorus (Figure 1F,G). The mRNA expression of the *receptor activator of nuclear factor kappa-Β ligand* (*RANKL*), *osteoprotegerin* (*OPG*), *bone morphogenetic protein 2* (*BMP2*), and *runt-related transcription factor 2* (*RUNX2*), which are bone metabolism-related cytokines, in the tibia of the 10^9^ CB group was not significantly different from that of the Con group (*p* > 0.05) (Figure 1H).

### 3.2. Effect of CB on Luhua Laying Hens

**Figure 2:**
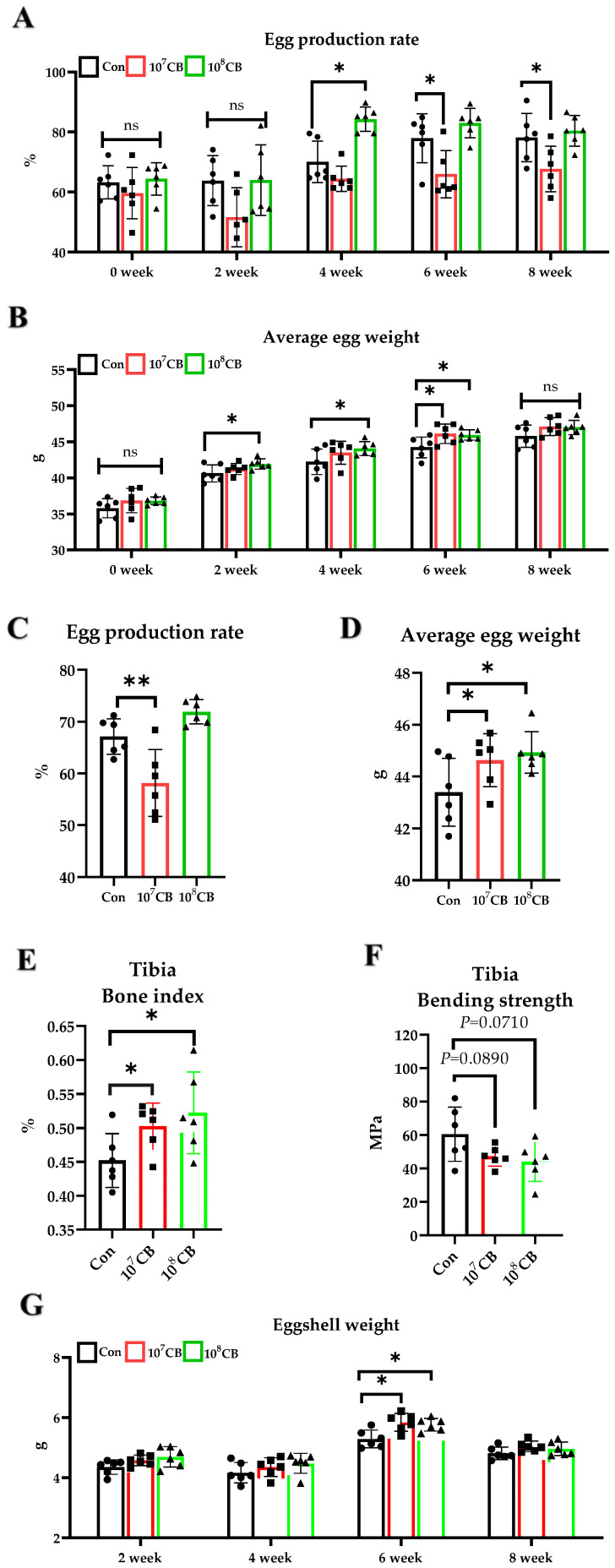
Effect of *Clostridium butyricum* (CB) on Luhua laying hens. (**A**,**B**) Changes in egg production rate and average egg weight of laying hens over time. (**C**) Egg production rate from 1–8 weeks. (**D**) Average egg weight from 1–8 weeks. (**E**–**G**) Tibia index, tibia bending strength, and eggshell weight. Con: Control group, fed basal diet; 10^7^ CB: basal diet supplemented with 1 × 10^7^ CFU/kg CB; 10^8^ CB: basal diet supplemented with 1 × 10^8^ CFU/kg CB. The data are presented as the mean ± SD., * *p* < 0.05, ** *p* < 0.01, ns, no difference.

The egg production rate and average egg weight were calculated every two weeks. Compared with the Con group, the egg production rate of the 10^7^ CB group was significantly decreased in the sixth and eighth weeks (*p* < 0.05), and that of the 10^8^ CB group was significantly increased in the fourth week (*p* < 0.05) (Figure 2A). The overall period egg production rate of the 10^7^ CB group was significantly lower than that of the Con group (*p* < 0.01), and there was no difference between the 10^8^ CB group and the Con group (*p* > 0.05) (Figure 2C). Compared with the Con group, the average egg weight of the 10^7^ CB group increased significantly in the sixth week (*p* < 0.05), and that of the 10^8^ CB group significantly increased in the second, fourth, and sixth weeks (*p* < 0.05) (Figure 2B). The overall period average egg weights of the 10^7^ CB group and the 10^8^ CB group were significantly higher than that of the Con group (*p* < 0.05) (Figure 2D). The feed intake and feed conversion ratio were not significantly affected by supplementation CB (Figure S1). Specific data are provided in the Supplementary File (Table S3). Compared with the Con group, the 10^7^ CB group showed a significant eggshell thickness increase in the eighth week of the experiment (*p* < 0.05). The 10^8^ CB group showed a significant Haugh unit decrease in the eighth week (*p* < 0.05) (Table 3). There were no significant differences in eggshell index between the groups, but interestingly, we found differences in eggshell weight. The eggshell weights of the 10^8^ CB and 10^7^ CB groups were significantly higher than that of the Con group during week six of the experiment (*p* < 0.05) (Figure 2G). The tibial index of the 10^7^ CB group and the 10^8^ CB group were significantly higher than the Con group (*p* < 0.05) (Figure 2E). However, the tibia bending strengths of the 10^7^ CB and 10^8^ CB groups were not significantly different from those of the Con group (*p* > 0.05) and even tended to decrease (Figure 2F). There were no significant differences in other assay data, and specific data are provided in the Supplementary File (Table S4).

### 3.3. Effect of CB on Hy-Line Brown Laying Hens

**Figure 3:**
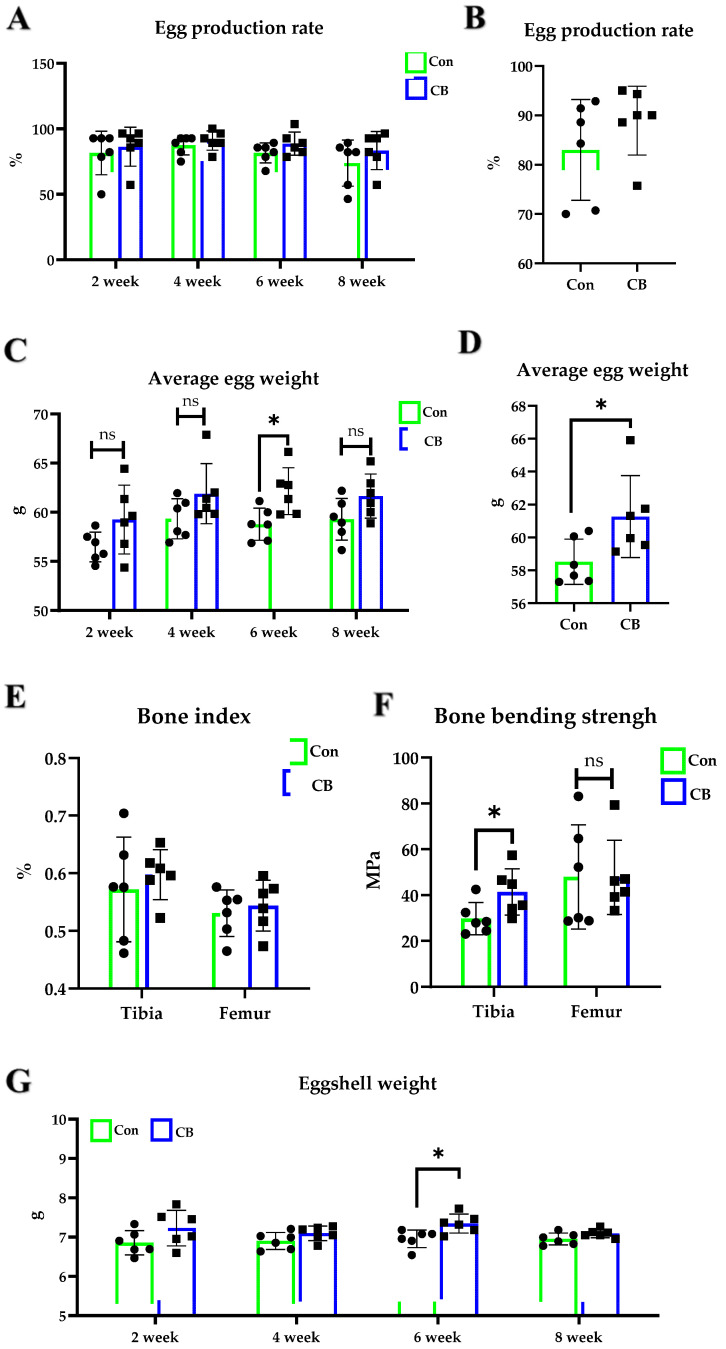
Effect of *Clostridium butyricum* (CB) on Hy-line Brown laying hens. (**A**,**B**) Changes in egg production rate and average egg weight of laying hens over time. (**C**) Egg production rate from 1–8 weeks. (**D**) Average egg weight from 1–8 weeks. (**E**) Tibia and femur index. (**F**) Bending strength of tibia and femur. (**G**) Eggshell weight. Con: Control group, fed basal diet; CB: basal diet supplemented with 1 × 10^8^ CFU/kg CB. The data are presented as the mean ± SD. * *p* < 0.05, ns, no difference.

There was no significant effect of 10^8^ CB treatment on the egg production rate (*p* > 0.05) (Figure 3A,B). The week six average egg weight of the 10^8^ CB group was higher than that of the Con group (*p* < 0.05) (Figure 3C). The overall period average egg weight of the 10^8^ CB group was significantly higher than that of the Con group (*p* < 0.05) (Figure 3D). The feed intake and feed conversion ratio were not significantly affected by supplementation CB (Figure S2). Specific data are provided in the Supplementary File (Table S5). Compared with the Con group, the egg shape index decreased significantly in the fourth (*p* < 0.001), sixth (*p* < 0.01), and eighth (*p* < 0.05) weeks. The Haugh unit increased significantly in the eighth week (*p* < 0.05) (Table 4). We also measured the eggshell weight and found that the eggshell weight of the 10^8^ CB group was significantly higher than that of the Con group in week six (*p* < 0.05) (Figure 3G). Adding 10^8^ CB to the feed had no significant effect on the tibia index and femur index (*p* > 0.05) (Figure 3E). The bending strength of the tibia in the 10^8^ CB group was significantly higher than in the Con group (*p* < 0.05), but there was no significant change in the bending strength of the femur (*p* > 0.05) (Figure 3F). There were no significant differences in other assay data, and specific data are provided in the Supplementary File (Table S6).

## 4. Discussion

To date, the role of probiotics as feed additives has been extensively studied and valued. Numerous studies have shown that adding probiotics to feed can improve nutrient use efficiency and animal growth performance [5,6]. This study mainly explored the effects of CB on the production performance and bone health of laying hens.

Many experiments have used broiler chickens as a model and demonstrated the effect of CB on promoting weight gain. Yu found that the dietary supplementation of 1 × 10^9^ CFU/kg CB increased weight gain in Cobb broilers [7]. Li determined that feed supplementation with 1 × 10^9^ CFU/kg CB could increase the body weight and feed intake of Arbor Acres broilers [8]. We used laying hens as a model and added 10^9^ CFU/kg of CB but did not obtain the same results. The reason for this may be related to the different growth rates of laying hens and broiler chickens and the slower growth and development of laying hens. Zhang found that feeding 10^9^ CFU/kg CB would reduce the cecal length of broiler chickens and affect the intestinal metabolic function of broiler chickens [9], but our research determined that CB had no significant effect on laying hens. The bone health of laying hens is inseparable from production performance. Medullary bone is a unique structure of laying hens and plays a vital role as a calcium storage reservoir for eggshells [10]. Calcium absorbed from food is deposited in the medullary bone, and during egg laying the calcium is reabsorbed and later deposited on the eggshell [11]. Several studies have shown the beneficial effects of CB on bone health in mice [12,13]. Tibia is an effective indicator for evaluating the bone size of poultry [14]. Our research results found that CB can significantly increase the tibia index of four-week-old green-shell laying hens. The changing trend of tibia BMD also proves the promoting effect of CB on tibia development. Higher BMD generally represents a higher calcium and phosphorus content and stronger bone hardness. CB has a certain tendency to promote tibial calcium but has no effect on phosphorus. Interestingly, our analysis found that CB did not significantly increase the flexural strength of the tibia. Bones are composed of osteocytes and inorganic and organic extracellular matrix. Osteocytes are mainly osteoclasts, which absorb bone tissue, and osteoblasts, which deposit bone tissue. RUNX2 and BMP2 are crucial for osteoblast differentiation and maturation [15]. They are key genes in the osteoblast bone formation process and have an important instructive role in bone formation. RANKL binds to its receptor RANK and monocyte osteoclast precursor cells to stimulate osteoclast differentiation and bone resorption capacity. The interaction between RANKL and RANK is regulated by osteoprotegerin (OPG) secreted by osteoblasts and inhibits osteoclastogenesis [16]. However, our research results did not show the effect of CB treatment on RANKL, OPG, BMP2, and RUNX2 mRNA expression. The body’s bones are regulated in many ways, and the tibial index increase reasons need to be further explored.

To confirm the effect of CB on egg production performance, we selected rigid mature Luhua laying hens and Hy-line Brown laying hens at the peak of egg production as experimental subjects. When using adult chickens as the experimental model, we reduced the amount of CB used in consideration of the cost of actual use. Wang’s research found that 1 × 10^8^ CFU/kg CB can increase the egg production rate and albumen height of 45-week-old Lingnan yellow-feathered breeder hens [17]. Obianwuna’s research found that 2 × 10^8^ CFU/kg CB can increase the average egg weight, shell thickness, shell strength, yolk color, albumen height, and Haugh units of 30-week-old Hy-line Brown laying hens [18]. Khogali’s research found that supplementing 1 × 10^8^ CFU/kg CB increased the egg production rate and average egg weight of 64-week-old Hy-line Brown laying hens [19]. In Lu’s study, the use of a higher dose of CB (1.2 × 10^9^ CFU/kg) had no significant effect on the production performance and egg quality of 22-week-old Jingfen No. 6 laying hens [20]. Our study found that adding 10^8^ CFU/kg CB was beneficial to the average egg weight and eggshell weight of Luhua laying hens and Hy-line Brown laying hens. Overall, 1 × 10^8^ CFU/kg CB is the dose with the best effect on promoting the production performance of laying hens. Interestingly for bone development, CB increased the tibia index of green-shell and Luhua laying hens but did not change their bone bending strength. However, CB had no effect on the bone index of Hy-line Brown laying hens but increased the tibia bending strength. This may be related to the different egg-laying stages of laying hens. The bones of laying hens are still in a rapid development stage during the brooding period and early laying period [21]. Forty-week-old Hy-line Brown laying hens are in the peak egg-laying stage, when the laying hens’ bone development is basically complete. The bone calcium metabolism of laying hens during the peak laying period is very intense [11], and the bones undergo intense resorption and formation every day. The mechanical strength of medullary bone is weaker than that of structural bone (cortical bone and cancellous bone) and does not contribute to bone strength [22]. During egg production, part of the calcium in the structural bones is also reabsorbed, which leads to a reduction in bone strength and fractures in laying hens [23]. The increase in bone flexural strength indicates that CB has a protective effect on the bones of laying hens during peak egg production.

## 5. Conclusions

In conclusion, this study shows that the promoting effect of CB on the production performance of laying hens is mainly reflected in increasing the average egg weight and eggshell weight of 26-week-old Luhua laying hens and 40-week-old Hy-line Brown laying hens. The effect of CB on bones is reflected in the improvement of tibial index of four-week-old green-shell laying hens and 26-week-old Luhua laying hens and the improvement in the tibial bending strength of 40-week-old Hy-line Brown laying hens. Our study found that supplementing feed with 10^8^ CFU/kg CB is a reasonable and effective dose. These findings contribute to a more comprehensive understanding of the effects of CB on bone development and production performance of laying hens, allowing for safer and more efficient use in poultry production. However, this study did not confirm the specific mechanism of CB promoting tibia development and average egg weight in laying hens. The mechanism of action of CB still needs to be further explored.

## Figures and Tables

**Table 1:** Composition of the experimental diet.

Green-Shell Laying Hens	
Items	Composition, %
Ingredients	
Corn	66
Soybean meal	29
Limestone	0
NaCl	0.3
Choline chloride (50%)	0.1
Premix ^1^	4.60
Total	100
Nutrient levels ^2^	
Metabolizable energy (Kcal/kg)	2900
Crude protein (%)	17.7
Lysine (%)	0.94
Methionine (%)	0.61
Calcium (%)	0.7
Available phosphorus (%)	0.47
Luhua Laying Hens	
Items	Composition, %
Ingredients	
Corn	65
Soybean meal	23
Limestone	7
NaCl	0.3
Choline chloride (50%)	0.1
Premix ^1^	4.60
Total	100
Nutrient levels ^2^	
Metabolizable energy (Kcal/kg)	2700
Crude protein (%)	15.0
Lysine (%)	0.77
Methionine (%)	0.53
Calcium (%)	3.20
Available phosphorus (%)	0.45
Hy-line Brown Laying Hens	
Items	Composition, %
Ingredients	
Corn	63
Soybean meal	24
Limestone	8
NaCl	0.3
Choline chloride (50%)	0.1
Premix ^1^	4.60
Total	100
Nutrient levels ^2^	
Metabolizable energy (Kcal/kg)	2700
Crude protein (%)	15.3
Lysine (%)	0.79
Methionine (%)	0.53
Calcium (%)	3.50
Available phosphorus (%)	0.45

^1^ The vitamin and mineral premix used the following quantities per kilogram of diet: vitamin A, 8800 IU; vitamin D3, 3300 IU; vitamin K, 2.2 mg; vitamin E, 16.5 IU; cholecalciferol, 2800 IU; riboflavin, 18 mg; niacin, 50 mg; pantothenic acid, 28 mg; biotin, 0.1 mg; folic acid, 0.6 mg; iron, 55 mg; selenium, 0.3 mg; copper, 5.5 mg; zinc, 88 mg; iodine, 1.7 mg; manganese, 88 mg; calcium, 5.7 g; and phosphorus, 3.3 g. ^2^ Nutrient levels are all calculated values.

**Table 2:** Primers for the qRT-PCR used in this study.

Gene Name	Genbank Number	Primer Position	Primer Sequences (5′→3′)
*RANKL*	NM001083361.2	Forward	TGTTGGCTCTGATGCTTGTC
		Reverse	TCCTGCTTCTGGCTCTCAAT
*OPG*	DQ098013.1	Forward	CGCTTGTGCTCTTGGACATT
		Reverse	GCTGCTTTACGTAGCTCCCA
*BMP2*	NM001398170.1	Forward	CCTTCGGAAGACGTCCTCAG
		Reverse	CTGAGTGCCTGCGGTACAGA
*RUNX2*	NM204128.1	Forward	TTTTTCCTGCCCGTATTCTG
		Reverse	GCTTGGTGCTGGAGAGTCTT
*β-actin*	L08165	Forward	GAGAAATTGTGCGTGACATCAAGG
		Reverse	CACCTGAACCTCTCATTGCCA

**Table 3:** Effect of *Clostridium butyricum* (CB) on egg quality of Luhua laying hens ^1^.

Items	Con	10^7^ CB	10^8^ CB	*p* =	F =
**8 Week**					
**Eggshell thickness** **, 0.01 mm**	30.31 ± 0.60 ^b^	33.3 ± 0.67 ^a^	31.26 ± 0.50 ^b^	0.0033	F (2,15) = 8.54
**Haugh unit**	68.13 ± 1.75 ^a^	68.37 ± 1.94 ^a,b^	61.98 ± 1.44 ^b^	0.0722	F (2,15) = 3.15

^1^ The data are presented as the mean ± SD. Con: Control group, fed basal diet; 10^7^ CB: basal diet supplemented with 1 × 10^7^ CFU/kg CB; 10^8^ CB: basal diet supplemented with 1 × 10^8^ CFU/kg CB. ^a,b^ Means sharing different letters in the same row are significantly different (*p* < 0.05).

**Table 4:** Effect of *Clostridium butyricum* (CB) on egg quality of Hy-line Brown layinghens ^1^.

Items	Con	CB	*p* =	F =
**2 Week**				
**4 Week**				
**Egg shape index, %**	1.3 ± 0.003 ^a^	1.27 ± 0.006 ^b^	0.0003	F (1,10) = 30.30
**6 Week**				
**Egg shape index, %**	1.3 ± 0.005 ^a^	1.27 ± 0.007 ^b^	0.0200	F (1,9) = 7.64
**8 Week**				
***Egg shape index, %***	1.3 ± 0.004 ^a^	1.28 ± 0.008 ^b^	0.0468	F (1,10) = 5.14
**Haugh unit**	79.88 ± 1.10 ^b^	84.90 ± 2.02 ^a^	0.0207	F (1,10) = 7.52

^1^ The data are presented as the mean ± SD. Con: Control group, fed basal diet; CB: basal diet supplemented with 1 × 10^8^ CFU/kg CB. ^a,b^ Means sharing different letters in the same row are significantly different (*p* < 0.05).

## Data Availability

Data are contained within the article and Supplementary Materials.

## Supplementary Materials

The following supporting information can be downloaded at: https://www.mdpi.com/article/10.3390/vetsci11040160/s1. Figure S1. Effects of *Clostridium butyricum* (CB) on feed intake and feed conversion ratio of Luhua layer hen. Figure S2. Effect of *Clostridium butyricum* (CB) on feed intake and feed conversion ratio of Hy-line Brown layer hens. Table S1. Effects of *Clostridium butyricum* (CB) on feed intake and growth of Green-shell layer hens. Table S2. Effect of *Clostridium butyricum* (CB) on production performance of Luhua layer hens. Table S3. Effect of *Clostridium butyricum* (CB) on production performance of Hy-line Brown layer hens. Table S4. Effect of *Clostridium butyricum* (CB) on egg quality of Luhua layer hens. Table S5. Effect of *Clostridium butyricum* (CB) on production performance of Hy-line Brown layer hens. Table S6. Effect of *Clostridium butyricum* (CB) on egg quality of Hy-line Brown layer hens.
