# Supplementary material for: Effects of Clostridium butyricum on Production Performance and Bone Development of Laying Hens
Source: Vet Sci. 2024 Apr 1;11(4):160. doi: 10.3390/vetsci11040160 (PMC11053732; doi:10.3390/vetsci11040160)
Supplement: Supplementary file 1 [file vetsci-11-00160-s001.zip › vetsci-2881226-supplementary.pdf]

Supplementary Materials:

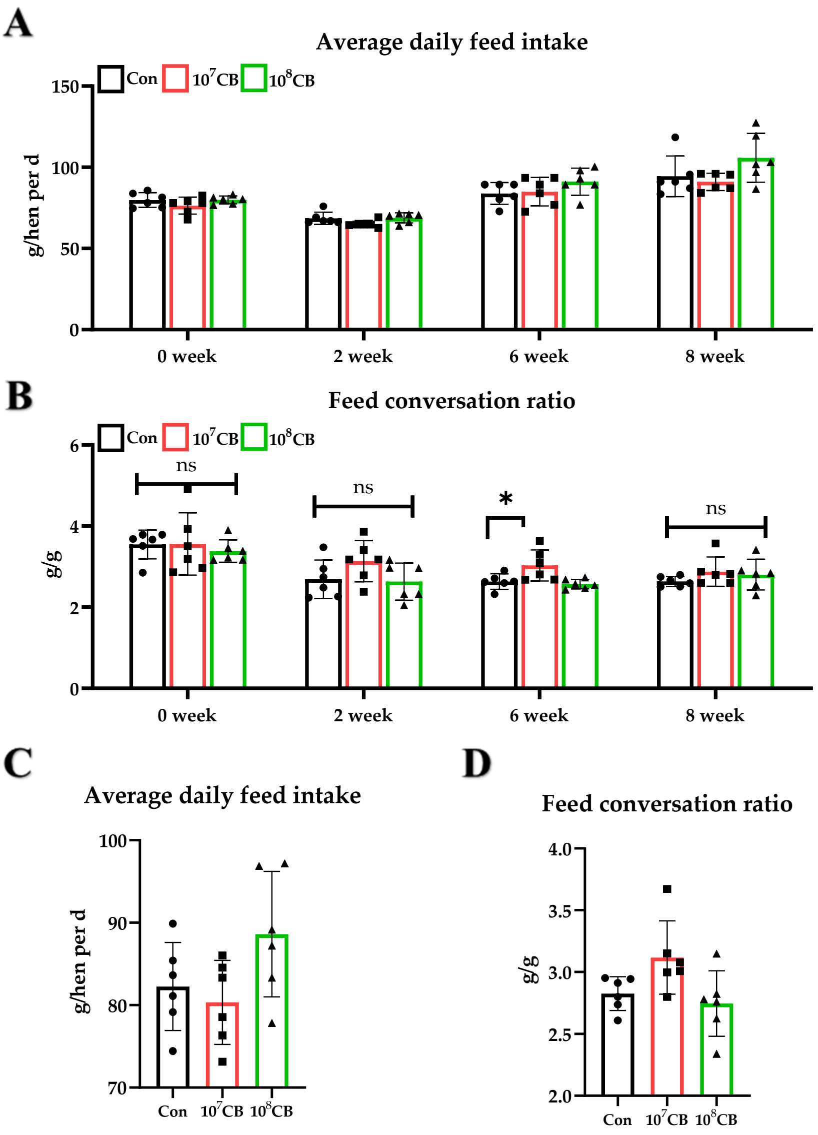

Figure S1. Effects of *Clostridium butyricum* (CB) on feed intake and feed conversion ratio of Luhua layer hens. (A, B) Changes in feed intake and feed conversion ratio of laying hens over time. (C) Feed intake from 1-8 weeks. (D)Feed conversion ratio from 1-8 weeks. Con: Control group fed basal diet; 10^7CB: basal diet supplemented with 1×10^7 CFU/kg CB; 10^8CB: basal diet supplemented with 1×10^8 CFU/kg CB. The data are presented as the mean ± SD. *, $P < 0.05$, ns, no difference.

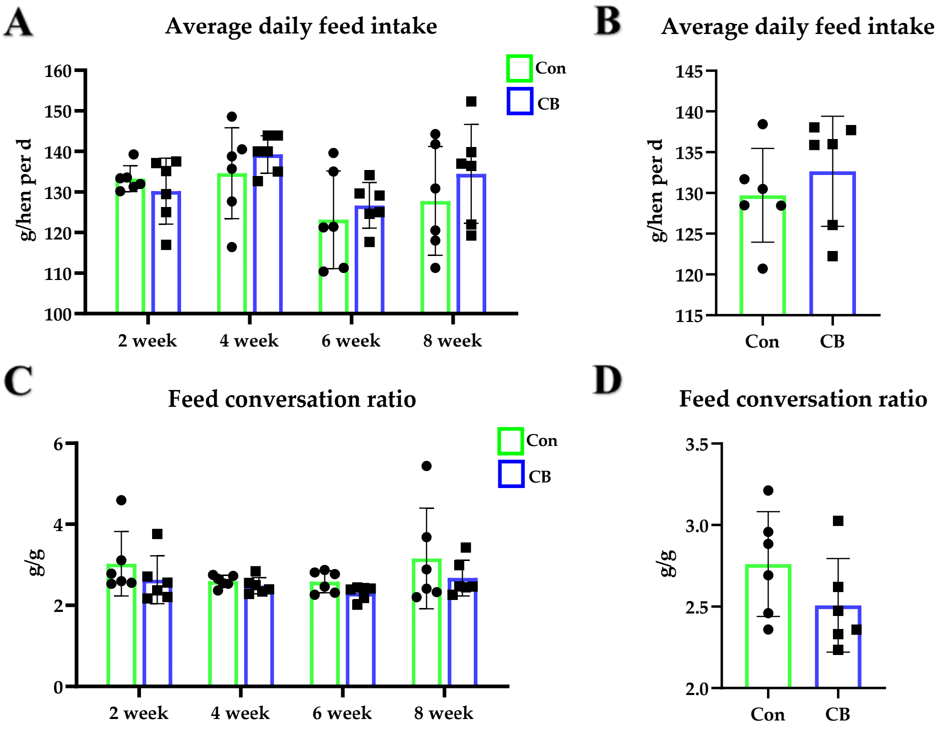

Figure S2. Effect of *Clostridium butyricum* (CB) on feed intake and feed conversion ratio of Hy-line Brown layer hens. (A, B) Changes in feed intake and feed conversion ratio of laying hens over time. (C) Feed intake from 1-8 weeks. (D) Feed conversion ratio from 1-8 weeks. Con: Control group fed basal diet; CB: basal diet supplemented with 1×10^8 CFU/kg CB. The data are presented as the mean ± SD.

Table S1. Effects of *Clostridium butyricum* (CB) on feed intake and growth of Green-shell layer hens [1]

Productive performance	Treatments			P =	F =
	Con	10^8CB	10^9CB		
Body weight (g) 28 days	305.75±5.72	302.13±4.85	305.38±5.93	0.8784	F(2,21) =0.13
Feed intake (g/hen/day) 0-28 day	207.16±0.86[a]	199.75±2.11[b]	199.61±1.97[b]	0.0206	F(2,9) =6.16

[1] The data are presented as the mean ± SD. Con: Control group, fed basal diet; 10^8CB: basal diet supplemented with 1×10^8 CFU/kg CB; 10^9CB: basal diet supplemented with 1×10^9 CFU/kg CB.

[a,b] Means sharing different letters in the same row are significantly different (P < 0.05).

Table S2. Effects of *Clostridium butyricum* (CB) on organ development of Green-shell layer hens [1].

	**Control**	**10^8CB**	**10^9CB**	***P*-value**	**F-value**
**Liver index, %**	3.02±0.17	2.71±0.07	2.81±0.06	0.1606	$F_{(2,21)}$ =2.00
**Spleen index, %**	0.27±0.03	0.30±0.03	0.28±0.02	0.8175	$F_{(2,21)}$ =0.20
**Bursa of Fabricius index, %**	0.48±0.03	0.56±0.08	0.45±0.03	0.2993	$F_{(2,21)}$ =1.28
**Thymus index, %**	0.8±0.061	0.95±0.153	0.843±0.051	0.5739	$F_{(2,21)}$ =0.57

**Duodenum length, cm**	18.59±0.6	17.83±0.7	17.43±0.5	0.3831	$F_{(2,21)}$ =1.00
**Jejunum length, cm**	37.78±1.9	36.36±1.8	34.71±0.8	0.4017	$F_{(2,21)}$ =0.95
**Ileum length, cm**	42.44±1.8	42.45±1.0	40.83±0.9	0.6045	$F_{(2,21)}$ =0.51
**Cecum length, cm**	9.26±0.34	9.25±0.21	9.31±0.35	0.9886	$F_{(2,21)}$ =0.01

[1] The data are presented as the mean ± SD. Con: Control group, fed basal diet; 10^8CB: basal diet supplemented with $1×10^8$ CFU/kg CB; 10^9CB: basal diet supplemented with $1×10^9$ CFU/kg CB.

Table S3. Effect of *Clostridium butyricum* (CB) on production performance of Luhua layer hens [1].

Productive performance	Treatments				
	Con	10^7CB	10^8CB	*P* =	F =
Egg production rate (%)					
0 week	63.24±2.25	59.67±3.48	64.43±2.20	0.4523	F(2,15) =0.84
2 week	63.84±3.39	51.64±4.02	63.99±4.80	0.0815	F(2,15) =2.98
4 week	70.08±2.84^b	64.48±1.74^b	84.28±1.74^a	0.0001	F(2,15)=22.68
6 week	77.93±3.32^a	65.96±3.20^b	82.97±2.00^a	0.0026	F(2,15) =9.05
8 week	78.17±3.27^a	67.74±3.08^b	80.40±2.09^a	0.0153	F(2,15) =5.59
overall period	67.13±1.40^a	58.15±2.63^b	71.92±0.97^a	0.0003	F(2,15)=14.95
Average egg weight (g)					
0 week	35.80±0.54	36.87±0.69	36.83±0.22	0.2920	F(2,15) =1.34
2 week	40.64±0.48^b	41.25±0.32^{ab}	41.93±0.30^a	0.0798	F(2,15) =3.01
4 week	42.25±0.72	43.50±0.65	44.08±0.40	0.1259	F(2,15) =2.39
6 week	44.24±0.59^b	46.12±0.54^a	45.97±0.29^a	0.0287	F(2,15) =4.54
8 week	45.80±0.63	47.13±0.50	47.00±0.40	0.1729	F(2,15) =1.98
overall period	43.39±0.53^b	44.63±0.42^{ab}	44.93±0.33^a	0.0562	F(2,15) =3.51
Average daily feed intake(g/hen per d)					
0 week	79.80±1.85	76.35±2.13	79.88±1.00	0.2881	F(2,15) =1.35
2 week	68.53±1.55	65.03±0.91	68.82±1.27	0.0946	F(2,15) =2.77
6 week	83.85±2.74	84.95±3.57	91.13±3.41	0.2655	F(2,15) =1.45
8 week	94.44±5.08	91.01±2.18	105.85±6.12	0.1024	F(2,15) =2.66
overall period	82.27±2.18^{ab}	80.33±2.08^b	88.60±3.10^a	0.0801	F(2,15) =3.00
Feed conversion ratio(g/g)					
0week	3.55±0.14	3.56±0.31	3.38±0.11	0.8051	F(2,15) =0.22
2 week	2.69±0.19	3.13±0.21	2.63±0.19	0.1756	F(2,15) =1.96
6 week	2.63±0.08^b	3.03±0.16^a	2.57±0.05^b	0.0138	F(2,15) =5.78
8 week	2.64±0.05	2.88±0.15	2.80±0.15	0.3984	F(2,15) =0.98
overall period	2.83±0.06^{ab}	3.12±0.12^a	2.75±0.11^b	0.0437	F(2,15) =3.89

[1] The data are presented as the mean ± SD. Con: Control group, fed basal diet; 10^7CB: basal diet supplemented with $1×10^7$ CFU/kg CB; 10^8CB: basal diet supplemented with $1×10^8$ CFU/kg CB.

[a,b] Means sharing different letters in the same row are significantly different ($P < 0.05$).

Table S4. Effect of *Clostridium butyricum* (CB) on egg quality of Luhua layer hens [1.]

Items	Con	10^7CB	10^8CB	*P* =	F =
**2Week**					
**Egg shape index, %**	1.32±0.009	1.32±0.035	1.29±0.02	0.5512	F(2,15) =0.62
**Eggshell thickness, 0.01mm**	31.71±1.2	32.43±0.83	33.04±0.82	0.5189	F(2,15) =0.69
**Eggshell index, %**	10.83±0.23	11.09±0.16	11.26±0.22	0.4251	F(2,15) =0.91
**Eggshell hardness, N**	37.55±1.91	35.53±1.58	39.48±1.13	0.2125	F(2,15) =1.72
**Albumen height, mm**	4.3±0.10	4.1±0.18	4.35±0.19	0.4989	F(2,15) =0.73
**Egg yolk color**	8.66±0.11	9.01±0.20	8.59±0.30	0.1930	F(2,15) =1.84
**Yolk Index, %**	31.73±0.52	28.83±1.33	30.92±0.65	0.3395	F(2,15) =1.16
**Haugh unit**	71.91±0.98	70.03±1.75	72.19±1.9	0.5704	F(2,15) =0.58
**4Week**					
**Egg shape index, %**	1.3±0.017	1.08±0.063	1.29±0.01	0.0739	F(2,15) =3.11
**Eggshell thickness, 0.01mm**	29.98±0.57	30.8±0.82	32.27±1.24	0.4521	F(2,15) =0.84
**Eggshell index, %**	9.71±0.16	9.82±0.23	9.95±0.18	0.6010	F(2,15) =0.53
**Eggshell hardness, N**	39.11±0.95	36.74±2.15	38.71±1.74	0.6341	F(2,15) =0.47
**Albumen height, mm**	5.14±0.28	4.56±0.28	4.59±0.20	0.1914	F(2,15) =1.85
**Egg yolk color**	8.56±0.14	9.12±0.36	9.12±0.47	0.5145	F(2,15) =0.69
**Yolk Index, %**	31.03±0.63	30.41±0.49	29.56±0.48	0.1014	F(2,15) =2.68
**Haugh unit**	76.49±2.21	69.52±2.33	71.63±1.59	0.0880	F(2,15) =2.87
**6Week**					
**Egg shape index, %**	1.3±0.013	1.32±0.015	1.3±0.09	0.0.5757	F(2,15) =0.57
**Eggshell thickness, 0.01mm**	30.67±0.073	31.61±0.7	31.31±0.79	0.7100	F(2,15) =0.35
**Eggshell index, %**	11.94±0.36	12.32±0.29	12.27±0.19	0.5484	F(2,15) =0.63
**Eggshell hardness, N**	37.22±2.66	37.44±1.88	39.85±1.76	0.7031	F(2,15) =0.36
**Albumen height, mm**	5.02±0.28	4.94±0.44	5.03±0.19	0.9196	F(2,15) =0.08
**Egg yolk color**	8.79±0.30	9.54±0.13	9.11±0.09	0.2112	F(2,15) =1.73
**Yolk Index, %**	30.04±0.50	29.64±0.56	29.49±0.43	0.3530	F(2,15) =1.12
**Haugh unit**	74.82±2.36	72.01±2.9	74.26±1.39	0.9463	F(2,15) =0.06
**8Week**					
**Egg shape index, %**	1.34±0.008	1.33±0.014	1.31±0.013	0.3542	F(2,15) =1.11
**Eggshell thickness, 0.01mm**	30.31±0.60[b]	33.3±0.67[a]	31.26±0.50[b]	0.0033	F(2,15) =8.54
**Eggshell index, %**	10.43±0.26	10.53±0.26	10.26±0.19	0.6912	F(2,15) =0.38
**Eeggshell hardness, N**	31.25±2.5	37.53±1.73	36.65±1.84	0.2443	F(2,15) =1.55
**Albumen height, mm**	4.22±0.21	4.46±0.19	3.73±0.13	0.1445	F(2,15) =2.21
**Egg yolk color**	8.85±0.09	8.72±0.18	8.79±0.06	0.3768	F(2,15) =1.04
**Yolk Index, %**	33.21±0.59	32.71±0.44	32.26±0.60	0.2735	F(2,15) =1.42
**Haugh unit**	68.13±1.75[a]	68.37±1.94[ab]	61.98±1.44[b]	0.0722	F(2,15) =3.15

[1] The data are presented as the mean ± SD. Con: Control group, fed basal diet; 10^7CB: basal diet supplemented with 1×10^7 CFU/kg CB; 10^8CB: basal diet supplemented with 1×10^8 CFU/kg CB.
[a,b] Means sharing different letters in the same row are significantly different ($P < 0.05$).

Table S5. Effect of *Clostridium butyricum* (CB) on production performance of Hy-line Brown layer hens [1].

	Treatments			
**Productive performance**	**Con**	**CB**	**p values**	**F values**
Egg production rate (%)				
2 week	81.55±6.80	86.31±6.08	0.6130	F(1,10) =0.27
4 week	87.50±3.02	91.07±3.02	0.4231	F(1,10) =0.70
6 week	81.55±3.12	88.69±3.62	0.1657	F(1,10) =2.24
8 week	73.81±7.18	83.33±5.95	0.3313	F(1,10) =1.04
overall period	82.98±4.17	88.93±2.84	0.2653	F(1,10) =1.39
Average egg weight (g)				
2 week	56.47±0.62	59.26±1.43	0.1035	F(1,10) =3.21
4 week	59.33±0.83[b]	61.90±1.25[a]	0.1185	F(1,10) =2.92
6 week	58.78±0.67	62.14±0.98	0.0175	F(1,10) =8.07
8 week	59.29±0.87	61.65±0.92	0.0903	F(1,10) =3.51
overall period	58.52±0.56	61.26±1.02	0.0399	F(1,10) =5.57
Average daily feed intake(g/hen per d)				
2 week	133.27±1.31	130.21±3.31	0.4096	F(1,10) =0.74
4 week	134.61±4.57	139.26±1.88	0.3698	F(1,10) =0.88
6 week	123.15±4.91	126.70±2.29	0.5284	F(1,10) =0.43
8 week	127.80±5.47	134.46±4.98	0.3889	F(1,10) =0.81
overall period	129.71±2.34	132.66±2.76	0.4345	F(1,10) =0.66
Feed conversionratio(g/g)				
2 week	3.029±0.32	2.632±0.24	0.3494	F(1,10) =0.96
4 week	2.598±0.06	2.485±0.08	0.2909	F(1,10) =1.24
6 week	2.581±0.11	2.313±0.07	0.0669	F(1,10) =4.22
8 week	3.158±0.51	2.676±0.18	0.3916	F(1,10) =0.80
overall period	2.761±0.13	2.507±0.12	0.1795	F(1,10) =2.08

[1] The data are presented as the mean ± SD. Con: Control group, fed basal diet; 10^7CB: basal diet supplemented with 1×10^7 CFU/kg CB; 10^8CB: basal diet supplemented with 1×10^8 CFU/kg CB; SB: basal diet supplemented with 1% sodium butyrate.
[a,b] Means sharing different letters in the same row are significantly different ($P < 0.05$).

Table S6. Effect of *Clostridium butyricum* (CB) on egg quality of Hy-line Brown layer hens [1].

Items	Con	CB	*P* =	F =
**2Week**				
**Egg shape index, %**	1.3±0.007	1.28±0.009	0.1077	F(1,10) =3.12
**Eggshell thickness, 0.01mm**	36.73±0.92	36.2±0.68	0.8303	F(1,10) =0.05
**Eggshell index, %**	12.05±0.17	11.85±0.15	0.8194	F(1,10) =0.05
**Eggshell hardness, N**	48.82±1.08	47.73±1.04	0.3781	F(1,10) =0.85
**Albumen height, mm**	6.33±0.32	7.36±0.46	0.0984	F(1,10) =3.32
**Egg yolk color**	7.82±0.11	7.12±0.28	0.0820	F(1,10) =3.74
**Yolk Index, %**	24.48±0.40	25.06±0.40	0.7165	F(1,10) =0.14
**Haugh unit**	79.10±2.23	83.87±4.06	0.4189	F(1,10) =0.71
**4Week**				

**Egg shape index, %**	1.3±0.003[a]	1.27±0.006[b]	0.0003	F(1,10) =30.30
**Eggshell thickness, 0.01mm**	35.44±0.49	34.65±0.4	0.4094	F(1,10) =0.74
**Eggshell index, %**	11.64±0.11	11.45±0.09	0.6228	F(1,10) =0.26
**Eggshell hardness, N**	48.14±1.03	46.73±0.8	0.7504	F(1,10) =0.11
**Albumen height, mm**	7.3±0.15	7.5±0.25	0.3880	F(1,10) =0.81
**Egg yolk color**	7.56±0.20	7.94±0.19	0.3797	F(1,10) =0.84
**Yolk Index, %**	26.58±0.32	26.34±0.16	0.4831	F(1,10) =0.53
**Haugh unit**	85.41±0.85	85.15±1.81	0.9409	F(1,10) =0.01
**6Week**				
**Egg shape index, %**	1.3±0.005[a]	1.27±0.007[b]	0.0200	F(1,9) =7.64
**Eggshell thickness, 0.01mm**	35.97±0.72	36.54±0.5	0.6945	F(1,10) =0.16
**Eggshell index, %**	12.21±0.14	11.78±0.09	0.3965	F(1,10) =0.76
**Eggshell hardness, N**	45.67±1.95	43.32±1.77	0.4948	F(1,10) =0.50
**Albumen height, mm**	7.17±0.22	7.98±0.22	0.0928	F(1,10) =3.45
**Egg yolk color**	7.75±0.45	6.71±0.19	0.0652	F(1,10) =4.29
**Yolk Index, %**	26.00±0.30	26.78±1.05	0.2955	F(1,9) =1.23
**Haugh unit**	84.32±1.73	87.75±1.58	0.3023	F(1,10) =1.18
**8Week**				
**Egg shape index, %**	1.3±0.004[a]	1.28±0.008[b]	0.0468	F(1,10) =5.14
**Eggshell thickness, 0.01mm**	34.23±0.56	34.27±0.4	0.9665	F(1,10) =0.00
**Eggshell index, %**	11.56±0.15	11.39±0.09	0.3018	F(1,10) =1.19
**Eggshell hardness, N**	44.98±1.36	40.98±1.39	0.1514	F(1,10) =2.41
**Albumen height, mm**	7.28±0.16	7.68±0.25	0.1015	F(1,10) =3.25
**Egg yolk color**	7.26±0.24	7.37±0.10	0.6359	F(1,10) =0.24
**Yolk Index, %**	27.55±0.30	26.69±0.18	0.3545	F(1,10) =0.94
**Haugh unit**	79.88±1.10[b]	84.90±2.02[a]	0.0207	F(1,10) =7.52

[1] The data are presented as the mean ± SD. Con: Control group, fed basal diet; CB: basal diet supplemented with 1×10^8 CFU/kg CB.

[a,b] Means sharing different letters in the same row are significantly different ($P < 0.05$).
